# Comorbidities and COPD severity in a clinic-based cohort

**DOI:** 10.1186/s12890-018-0684-7

**Published:** 2018-07-16

**Authors:** Chantal Raherison, El-Hassane Ouaalaya, Alain Bernady, Julien Casteigt, Cecilia Nocent-Eijnani, Laurent Falque, Frédéric Le Guillou, Laurent Nguyen, Annaig Ozier, Mathieu Molimard

**Affiliations:** 1 Univ. Bordeaux, Inserm, Bordeaux Population Health Research Center, team EPICENE, UMR 1219, F-33000 Bordeaux, France; 2 Pole cardiothoracique, Respiratory Diseases Department, CHU de Bordeaux, F-33000 Bordeaux, France; 3 Rehabiliation Center, Cambo-les-Bains, France; 4 Pneumology Clinic, St Medard en Jalles, France; 5 General Hospital, Bayonne, France; 6 Pneumology Clinic, Bordeaux, France; 7 Pneumology Clinic, La Rochelle, France; 8 Pneumology Clinic, St Augustin, Bordeaux, France; 9 U1219 Pharmaco-epidemiology, Bordeaux University, Bordeaux, France; 10 Univ. Bordeaux, Inserm, Bordeaux Population Health Research Center, team EPICENE, UMR 1219, 146 rue Leo Saignat, 33076 Cedex Bordeaux, France

**Keywords:** COPD, Comorbidities, Cluster analysis, Management

## Abstract

**Background:**

Chronic obstructive pulmonary disease (COPD) is an important cause of morbidity and mortality around the world. The aim of our study was to determine the association between specific comorbidities and COPD severity.

**Methods:**

Pulmonologists included patients with COPD using a web-site questionnaire. Diagnosis of COPD was made using spirometry post-bronchodilator FEV1/FVC < 70%. The questionnaire included the following domains: demographic criteria, clinical symptoms, functional tests, comorbidities and therapeutic management. COPD severity was classified according to GOLD 2011. First we performed a principal component analysis and a non-hierarchical cluster analysis to describe the cluster of comorbidities.

**Results:**

One thousand, five hundred and eighty-four patients were included in the cohort during the first 2 years. The distribution of COPD severity was: 27.4% in group A, 24.7% in group B, 11.2% in group C, and 36.6% in group D. The mean age was 66.5 (sd: 11), with 35% of women**.** Management of COPD differed according to the comorbidities, with the same level of severity. Only 28.4% of patients had no comorbidities associated with COPD. The proportion of patients with two comorbidities was significantly higher (*p* < 0.001) in GOLD B (50.4%) and D patients (53.1%) than in GOLD A (35.4%) and GOLD C ones (34.3%). The cluster analysis showed five phenotypes of comorbidities: cluster 1 included cardiac profile; cluster 2 included less comorbidities; cluster 3 included metabolic syndrome, apnea and anxiety-depression; cluster 4 included denutrition and osteoporosis and cluster 5 included bronchiectasis. The clusters were mostly significantly associated with symptomatic patients i.e. GOLD B and GOLD D.

**Conclusions:**

This study in a large real-life cohort shows that multimorbidity is common in patients with COPD.

**Electronic supplementary material:**

The online version of this article (10.1186/s12890-018-0684-7) contains supplementary material, which is available to authorized users.

## Background

COPD has emerged as the most important respiratory disease worldwide. The epidemiology of COPD had changed in recent years, with more women affected [1], fewer old subjects and more medications available for health providers.

To improve the management of COPD and take into account the heterogeneity of the disease, the Global Obstructive Lung Disease Initiatives [2] proposed a new classification in 2011 that takes into account respiratory symptoms, the burden of exacerbations and lung function.

Comorbidities in COPD have received considerable attention as COPD patients frequently suffer from comorbidities such as cardiovascular and cerebrovascular disease, lung cancer and diabetes, with a significant impact on mortality that was termed by Divo et al. known as the “comorbidome” [3]. They constructed a comorbidity index (COTE index) based on 12 comorbidities that seem to negatively influence survival. However, the use of indexes like the COTE and the BODE [4] in clinical practice needs to be clarified. The validity of the COTE has been questioned since patients with GOLD B seem to have worse survival than patients with GOLD C, because of the particular heart disease found in a very large population study in Copenhagen area [5]. Some authors suggest that the presence of comorbidities should influence the relationship between the GOLD score and lung function measurements, the former perhaps being more representative of morbidity than of COPD severity [6].

The distribution and the type of comorbidities seem to vary between studies, except for cardiovascular disease which seems to be stable across them [7]. The complexity of COPD was reported in the Eclipse cohort, suggesting that COPD includes several different phenotypes when taking into account clinical parameters, survival, hospitalization, comorbidities and systemic inflammation [8]. Recently, in a complex analysis using network analysis, Divo et al. showed that comorbidities in COPD do not occur by chance [9].

In 213 patients included in a rehabilitation center, Vanfleteren et al. identified 13 comorbidities and five comorbidity clusters: less comorbidity, cardiovascular, cachectic, metabolic and psychological [10]. However, little is known about the reproducibility of these comorbidity phenotypes in COPD patients in real life and their association with COPD severity. Recently, the ATS/ERS consensus statement recommended that studies be performed either to confirm or rule out an association between specific comorbidities and COPD [11]. In an ongoing prospective observational cohort of outpatients with COPD followed up by pulmonologists, the aim of our study was to determine the association between specific comorbidities and COPD severity.

## Methods

### Study design and population

The Palomb cohort is an ongoing, prospective, multicenter, observational study of subjects with COPD recruited in pulmonary clinics in South-West of France in a real-life setting since the first January 2014 and followed up for 3 years (Additional file 1).

The CNIL (National Data Protection and Privacy Commission) and the CCTIRS (Advisory Committee for Data Processing in Health Research) approved the study, and informed consent was obtained before enrollment. The authors had asked the local ethics committee for feedback regarding the need for ethical clearance for such a retrospective analysis, and were advised that this was not warranted.

Consent for publication statement is not applicable as no personal information is provided in this manuscript.

Between January 2014 and February 2016, *n* = 1584 patients were enrolled in the study by pulmonologist and followed up in everyday practice, the data was obtained using a web-site questionnaire fulfilled by the pulmonologist on a secure platform and with specific agreement obtained for the storage of health data.

The inclusion criterion was a diagnosis of COPD on the basis of a lung function test according to the ATS/ERS standards [12] and made using spirometry with post-bronchodilator FEV1/FVC < 70%. Patients were excluded if they didn’t have the lung function criteria of COPD.

### Measurements

The website questionnaire included the following domains: demographic criteria (age, gender), smoking habits, clinical symptoms (mMRC dyspnea, chronic cough, exacerbations during past 12 months), body mass index (BMI) [4], lung function, comorbidities and therapeutic management (vaccinations, pulmonary rehabilitation, smoking cessation and prescribed inhaler medication).

### Comorbidities

Comorbidities (*n* = 19) were recorded systematically in a standardized manner by the pulmonologist. The diagnosis of comorbidity was assessed first by patient report then confirmed by either reviewing the patient’s medication list or when complementary tests were available in medical records. Bronchiectasis was recorded by clinical and/or radiologic criteria, as usual in clinical practice.

### COPD severity

Severity of COPD was classified as A,B,C or D according to GOLD 2011 [2]: 1) high/low symptoms using the mMRC dyspnea score < or ≥ 2; 2) the severity of airflow limitation [13]; and 3) the number of exacerbations per year. Despite the recent publication of GOLD 2017, GOLD 2011 was chosen because this classification was used by the pulmonologist in clinical practice during the study.

### Statistical analysis

Analysis of variance was used for continuous variables and Chi-squared tests were used for categorical variables. We performed a univariate analysis between comorbidities and COPD severity (GOLD 2011). We decided to retain for further analysis only the comorbidities that were significantly more frequent in more severe COPD stages (B,C,D) than in the mild stage (A). Then we performed a principal component analysis and a non-hierarchical (K-means) cluster analysis to describe the cluster of comorbidities. To better define the number of appropriate clusters, we used three statistical methods: the scree plot method, the percentage of variance explained, and the Kaiser-Guttman method.

Lastly, we performed a cluster analysis (ward method) to ensure stability of the different clusters. All analyses were performed with SAS software version 9.4. All statistical tests were two sided, with *P* < 0.05 considered to indicate statistical significance.

## Results

### Patients’ characteristics

A total of 1584 patients were included in the cohort during the first 2 years.

The GOLD 2011 distribution was as follows: 27.4% in group A, 24.7% in group B, 11.2% in group C, and 36.6% in group D, with no significant gender difference. The mean age was 66.5 (sd: 11), with 30% of women. Clinical symptoms, exacerbations, lung function and management according to COPD severity are presented in Table 1. 28.7% of the patients had had 2 or more exacerbations during the past year.

**Table 1:** Description of 1584 subjects with COPD according to GOLD 2011 Classification (frequency of each variable by COPD severity)

	A *N* = 435	B *N* = 391	C *N* = 178	D *N* = 580	*p*
Males, %	68.5	62.6	62.9	64.6	0.3006
Age, yr (SD)	63.2(10.4)	69(10.8)	63.6(10.6)	68.2(11)	0.0006
BMI, (kg/m2), % < 21					0.0001
(underweight) [21–26[(normal)	12.4	13.3	21.3	19.6	
[26–29[(overweight)	37.7	30.2	33.2	36.4	
> 29 (obese)	21.8	21.2	22.5	17.6	
	28.3	35.3	23	26.4	
FEV1, % pred					0.0001
> 80%	109(25)	55(14)	26(14.6)	13(2.2)	
50–80%	326(75)	336(86)	62(34.9)	141(24.3)	
30–50%	–	–	85(47.8)	328(56.5)	
< 30%	–	–	5(2.8)	98(16.9)	
mMRC 0–1, *n*(%)	435(100)	0	178(100)	0	0.0001
mMRC > =2, *n*(%)	0	391(100)	0	580(100)	0.0001
Chronic cough, *n*(%)	190(43.6)	222(56.7)	96(53.9)	402(69.3)	< 0.0001
Current smokers, *n*(%)	170(39.8)	131(34.5)	68(39.5)	195(34.5)	0.2394
0–1 exacerbation previous year, *n*(%)	435(100)	391(100)	71(40)	231(39.8)	0.0001
> = 2 exacerbations, previous year, *n*(%)	0	0	107(60)	349(60.2)	0.0001
Pulmonary rehabilitation, *n*(%)	12(2.7)	23(5.8)	10(5.6)	97(16.7)	< 0.0001
Smoking cessation, *n*(%)	35(8.0)	23(5.8)	26(14.6)	58(10)	0.0053
SABA, %	28.7	44.2	37	47	< 0.0001
LABA, or LAMA, %	53.1	72.1	66.8	71.2	< 0.0001
ICS and LABA, %	18.1	24.8	24.2	39	< 0.0001
Annual influenza vaccination, *n*(%)	166(38.1)	230(58.8)	93(52.2)	410(70.6)	< 0.0001
Pneumococcal vaccination, *n*(%)	141(32.4)	204(52.1)	100(56.1)	379(65.3)	< 0.0001

Abbreviations: *SABA* short-acting bronchodilators *LABA* long-acting bronchodilators. *LAMA* long-acting muscarinic antagonist

The onset of symptoms (i.e. cough, exacerbations or dyspnea) occurred between 25 and 49 years in 6% of the patients, 49–59 years in 20%, 59–69 years in 35%, 69–79 years in 25.3% and after 79 years in 13.26%.

16% had a BMI below 21 kg/m2, 34.8% between 21 and 26 kg/m2, 20% were overweight and 28.7% were obese (BMI > 29 kg/m2). BMI was significantly associated with severity of dyspnea and age (data not shown).

Prescription of pulmonary rehabilitation was rather infrequent in the whole population and was significantly more frequent in GOLD D patients (Table 1). By contrast, influenza vaccination was more frequent in GOLD B and D patients than pneumococcal vaccination, which increased with COPD severity (trend).

### Frequency of comorbidities

**Fig. 1:**
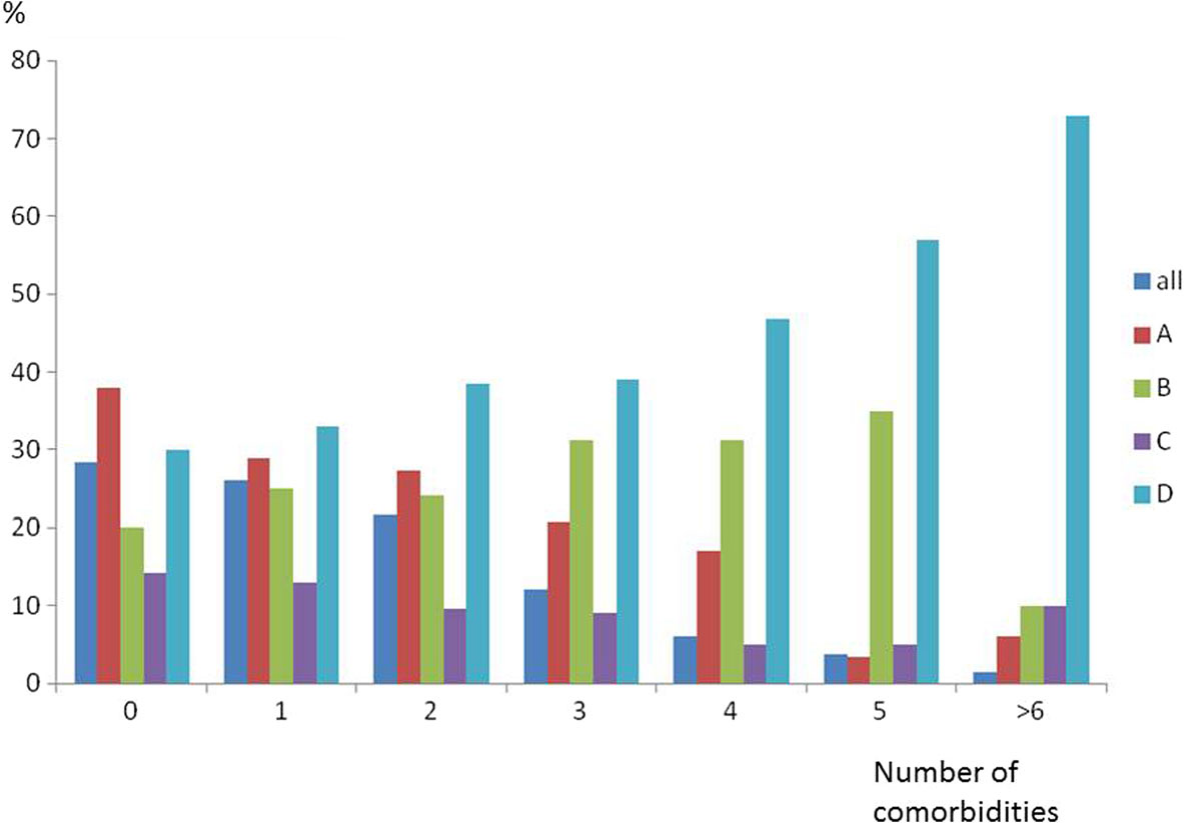
Frequency of COPD severity by number of comorbidities

Only 28.4% of patients had no comorbidities associated with COPD, whatever the severity of their COPD. The number of comorbidities by COPD severity is shown in Fig. 1, the number of comorbidities increased with severity of COPD. Cardiac comorbidities were more frequent in men whereas anxiety-depression and osteoporosis were more frequent in women.

Hypertension, ischemic cardiopathy, heart rhythm disorder and left cardiac insufficiency were significantly higher in overweight and obese subjects (*p*: 0.0001).

Prevalence of obstructive apnea syndrome (OAS) was higher in group A. Hypertension, OAS, dyslipidemia, ischemic cardiopathy and heart rhythm disorder were more frequent in groups B and D than in groups A and C. The frequencies of all comorbidities for COPD stage are presented in Table 2.

**Table 2:** Frequency of comorbidities in 1584 subjects with COPD according to GOLD 2011 Classification (frequency of each variable by COPD severity)

	A N = 435	B N = 391	C N = 178	D N = 580	*p*
Hypertension	139(31.9)	173(44.2)	52(29.2)	237(40.8)	0.0001
Obstructive apnea syndrome	118(27.1)	75(19.2)	23(12.9)	63(10.9)	0.0001
Dyslipidemia	97(22.3)	114(29.2)	37(20.8)	143(24.7)	0.072
Cancer, all causes	79(18.2)	70(17.9)	20(11.2)	90(11.5)	0.14
Ischemic cardiopathy	59(12.5)	99(25.3)	30(16.8)	127(21.9)	0.0001
Past asthma	47(10.8)	48(12.3)	19(10.7)	71(12.3)	0.84
Depression	43(9.9)	50(12.8)	15(8.4)	113(19.5)	0.0001
Anxiety	30(6.9)	70(17.9)	23(12.9)	154(26.5)	0.0001
Heart rhythm disorder	41(9.4)	65(16.6)	24(13.4)	86(14.8)	0.016
Diabetes	41(9.4)	51(13)	18(10.1)	88(15.2)	0.035
Undernutrition, BMI < 21 kg/m2	1(0.2)	3(0.7)	4(2.2)	25(4.3)	0.0001
Osteoporosis	2(0.4)	23(5.8)	1(0.5)	35(6.0)	0.0001
Atheroma	20(4.6)	27(6.9)	9(5.0)	51(8.8)	0.048
Left cardiac insufficiency	8(1.8)	21(5.3)	12(6.7)	50(8.6)	0.0001
Vascular Stroke	16(3.7)	22(5.6)	5(2.8)	20(3.5)	0.26
Pulmonary hypertension	3(0.7)	7(1.8)	1(0.6)	26(4.5)	0.0002
Metabolic syndrome	10(2.3)	16(4.1)	5(2.8)	27(4.7)	0.21
Rhinitis/rhinosinusitis	8(3.2)	14(7)	7(7.8)	19(7.4)	0.16
Bronchiectasis	7(3.9)	31(4.1)	34(9.2)	6(6.4)	0.004

We have identified 13 comorbidities which were more frequent in higher COPD stages (B,C,D) than in the mild stage (A). OAS was more frequent in mild stage (A) than in others.

Anxiety and depression was higher in groups D and B than in the other groups. Undernutrition was higher in group D. Osteoporosis was higher in groups B and D.

### Number of comorbidities

In the group of patients with one comorbidity, 36.7% had hypertension, 11.8% had Obstructive Syndrome Apnea (OSA), 10.4% had depression and 10% had ischemic cardiopathy. The proportion of patients with two comorbidities was significantly higher (*p* < 0.001) in GOLD B (50.4%) and D patients (53.1%) than in GOLD A (35.4%) and C ones (34.3%). The median of comorbidities was 1.6 (box plot) in the whole sample.

The proportion of GOLD B and D patients, increased significantly with the number of comorbidities, particularly among those with more than two comorbidities (Fig. 1). The number of comorbidities was higher in GOLD B and D patients (Table 3). The number of patients with two comorbidities or more was significantly higher in patients GOLD B and D patients than in GOLD A and C ones (Table 4).

**Table 3:** Number of comorbidities n (%) by COPD severity (frequency of Number of comorbidities in each COPD severity stage)

Number of comorbidities	A N = 435	B N = 391	C N = 178	D N = 580	*p*
0	160 (36.7)	90(23)	64(35.9)	136(23.4)	< 0.0001
1	121(27.8)	104(26.6)	53(29.8)	136(23.4)	< 0.0001
2	94(21.6)	83(21.2)	33(18.5)	132(22.7)	< 0.0001
3	40(9.2)	60(15.3)	17(9.5)	75(12.9)	< 0.0001
4	16(3.7)	30(7.7)	5(2.8)	45(7.8)	< 0.0001
5	2(0.4)	21(5.4)	3(1.7)	34(5.9)	< 0.0001
6 and more	2(0.4)	3(0.7)	3(1.7)	22(2.1)	< 0.0001

**Table 4:** Comorbidities frequency (> = 2 vs 0–1)by COPD severity (statistical test to compare the distribution of comorbidity frequency in each severity stage)

Comorbidity frequency	GOLD 2011				*p* value
	A N = 435	B N = 391	C N = 178	D N = 580	
*0–1864(54.55%)*	281(32.5%)	194(22.4%)	117(13.5%)	272(31.4%)	< 0.0001
*≥2720(45.45%)*	154(21.3%)	197(27.3%)	61(8.4%)	308(42.7%)	

### Management of COPD according to number of comorbidities

In GOLD A and B patients, prescription of treatment as needed and regular treatment did not differ according to the number of comorbidities (Table 5), unlike for GOLD C and D patients. In GOLD C patients, LABA were more frequently prescribed in those with comorbidities than in those without. In GOLD D patients, SABA was prescribed significantly more frequently in those with comorbidities. Pulmonary rehabilitation and vaccination were prescribed significantly more in GOLD B and D patients with comorbidities than in those with the same degree of severity but without comorbidities. Finally, smoking cessation was prescribed significantly more in GOLD C and D patients with comorbidities.

**Table 5:** COPD treatment by number of comorbidities and COPD severity (P value refers to compare each pharmacologic treatment according to number of comorbidities in each severity stage)

Number of comorbidities	A N = 435			B N = 391			C N = 178			D N = 580		
	0–1 *N* = 281	> = 2 *N* = 154	p	0–1 *N* = 194	> = 2 *N* = 197	p	0–1 *N* = 117	> = 2 *N* = 61	p	0–1 *N* = 272	> = 2 *N* = 308	*p*
SABA, n(%)	83(29.5)	42(27.2)	0.61	81(41.7)	92(46.7)	0.32	46(39.3)	20(32.8)	0.39	94(34.5)	179(58.2)	< 0.0001
LABA or LAMA, *n*(%)	152(54.0)	79(51.3)	0.57	139(71.6)	143(72.6)	0.83	72(61.5)	47(77.0)	0.03	186(68.4)	227(73.7)	0.15
ICS and LABA, *n*(%)	53(18.9)	26(16.9)	0.60	55(28.3)	42(21.3)	0.10	31(26.5)	12(19.7)	0.31	102(37.5)	124(40.3)	0.49
LABA and LAMA, *n*(%)	30(10.7)	23(14.9)	0.02	26(13.4)	38(19.3)	0.01	18(15.4)	19(31.1)	0.02	45(16.5)	94(30.5)	0.01
LABA and LAMA and ICS, *n*(%)	1(0.35)	1(0.64)	0.35	2(1.03)	2(1.01)	0.35	2(1.7)	2(3.2)	0.35	8(2.9)	21(6.8)	0.02
Pulmonary Rehabilitation, *n*(%)	8(2.8)	4(2.6)	0.87	6(3.9)	17(8.6)	0.02	7(6)	3(5)	0.76	31(11.4)	66(21.4)	0.0012
Annual influenza vaccination, *n*(%)	105(37.4)	61(39.6)	0.64	95(49)	135(68.5)	< 0.0001	60(51.3)	33(54.1)	0.72	167(61.4)	243(79)	< 0.0001
Pneumococcal vaccination, *n*(%)	85(30.2)	56(36.3)	0.19	81(41.7)	123(62.4)	< 0.0001	65(55.5)	35(57.3)	0.81	145(53.3)	234(76)	< 0.0001
Smoking cessation, *n*(%)	24(8.5)	11(7.14)	0.60	13(6.7)	10(5.0)	0.49	13(11.1)	13(21.3)	0.06	17(6.2)	41(13.3)	0.0047

### Comorbidity clusters

The cluster analysis showed five phenotypes of comorbidities: cluster 1 included cardiac profile; cluster 2 included less comorbidity; cluster 3 included metabolic syndrome, apnea and anxiety-depression; cluster 4 included cachectic and osteoporosis and cluster 5 included mainly bronchiectasis. The label of each cluster was given, comparing the prevalence of comorbidity in the whole population with prevalence of comorbidity in each cluster (Table 6).

**Table 6:** Prevalence of comorbidities in the five clusters

comorbidities (% in the whole population)	Cluster1 *N* = 360	Cluster2 *N* = 430	Cluster3 *N* = 233	Cluster4 N = 327	Cluster5 *N* = 234
OSA (17.6)	69(24.7)	36(12.9)	74(26.5)	60(21.5)	40(14.3)
Bronchiectasis (5.1)	17(21)	6(7.4)	20(24.7)	17(21)	21(25.9)
Left cardiac insufficiency (5.7)	19(20.9)	17(18.7)	23(25.3)	16(17.6)	16(17.6)
Hypertension (37.9)	130 (21.6)	98(16.3)	164 (27.3)	136 (22.6)	73(12.1)
Heart rhythm disorder (13.64)	48(22.2)	31(14.4)	54(25)	51(23.6)	32(14.8)
Atheroma (6.76)	23(21.5)	12(11.2)	35(32.7)	18(16.8)	19(17.8)
Pulmonary hypertension (2.3)	15(40.5)	4(10.8)	8(21.6)	5(13.5)	5(13.5)
Diabetes (12.5)	47(23.7)	26(13.1)	56(28.3)	51(25.8)	18(9.1)
Depression (13.95)	46(20.8)	33(14.9)	66(29.9)	44(19.9)	32(14.5)
Anxiety (17.5)	62(22.4)	15(5.4)	87(31.4)	67(24.2)	46(16.6)
Undernutrition (2)	10(30.3)	1(3)	8(24.2)	10(30.3)	4(12.1)
Ischemic cardiopathy (19.9)	61(19.4)	46(14.6)	83(26.3)	72(22.9)	53(16.8)
Osteoporosis (3.8)	13(21.3)	3(4.9)	14(23)	21(34.4)	10(16.4)

The different clusters were distributed in the four stages of COPD severity, however cardiac cluster was more frequent in patients with GOLD B. Cluster with less comorbidity was more frequent in patient GOLD C and A. Metabolic syndrome was more frequent in GOLD C and D. Cachectic and osteoporotic profile were most frequent in GOLD B and D. Lastly, bronchiectasis profile was more frequent in patient GOLD D (Table 7).

**Table 7:** Distribution of comorbidities cluster by COPD severity

Clusters	GOLD 2011				
	A N = 435	B N = 391	C N = 178	D N = 580	*p* value
Cluster 1 Cardiac (22.7%)	107(24.6)	102(26)	33(18.5)	118(20)	< 0.0001
Cluster 2 less comorbidity (27%)	137(31.5)	96(24.5)	57(32)	140(24.1)	< 0.0001
Cluster 3 metabolic, apneic and anxiety-depression (14.7%)	56(12.8)	39(9.9)	41(23)	97(16)	< 0.0001
Cluster 4 Cachectic and osteoporosis (20.6%)	70(16)	101(25.8)	22(12.3)	134(23)	< 0.0001
Cluster 5 bronchiectasis (14.7%)	65(14.9)	53(13.5)	25(14)	91(15.6)	< 0.0001

## Discussion

This study sought to determine whether comorbidities were associated with COPD severity in a clinic-based cohort of COPD patients, mostly GOLD A and B, followed up by pulmonologists. Only 28.4% of patients had no associated comorbidities. Fourteen comorbidities were significantly different with COPD severity. In this large population of patients, the median number of comorbidities was two.

Hypertension, OSA, dyslipidemia, ischemic cardiopathy and heart rhythm disorder were more frequent in GOLD B and D patients than in the other groups, as were anxiety and depression. Undernutrition was the most frequent in GOLD D patients and osteoporosis was the most frequent in GOLD B and D subjects. The number of comorbidities was the highest in GOLD B and D patients. Even when the severity of symptoms was similar, the management of COPD seemed to be different according to whether patients had comorbidities or not. Finally, five clusters of comorbidities were established, the most frequent being the cluster with cardiovascular disease and obstructive apnea syndrome.

Our approach was to analyze the relationship between these comorbidities and COPD severity by using three different approaches: the impact of the number of comorbidities, the univariate association between the comorbidities and COPD severity and cluster analysis to determine the association between the comorbidities i.e. to establish the existence of different phenotypes. Our findings are consistent with previous publications reporting a high prevalence of comorbidity in COPD, particularly cardio-vascular disease. Chen et al. [7] in a large review reported that compared with the non-COPD population, patients with COPD were more likely to be diagnosed with cardiovascular disease (odds ratio [OR] 2·4; 95% CI 2·02–3·00; *p* < 0·0001), including a two- to five-fold higher risk of ischemic heart disease, cardiac dysrhythmia, heart failure, diseases of the pulmonary circulation, and arterial disease. Additionally, patients with COPD reported hypertension more often (OR 1·3, 95% CI 1·1–1·5; *p* = 0·0007), diabetes (1·3, 1·2–1·5; p < 0·0001], and ever smoking (4·2, 3·2–5·6; *p* < 0·0001). Divo et al. found in their cohort that cardiovascular disease was highly associated with the risk of mortality, but that the highest risk of mortality was associated with anxiety [3]. However, we found a high prevalence of OAS, probably owing to the overlap syndrome as reported by Soler et al. [14]. By contrast, OAS was the most frequent comorbidity is GOLD A and B patients although it seemed to be associated with moderate-to-severe COPD. It is essential to diagnose OAS in patients with COPD as patients with overlap syndrome who are not treated with CPAP have a higher mortality [15].

In our cohort, 14 comorbidities were significantly associated with COPD severity. Hypertension, OSA, dyslipidemia, ischemic cardiopathy, and heart rhythm disorder were more frequent in GOLD B and D compared to GOLD A and C. Anxiety and depression was higher in GOLD D and B, compared to the other groups of severity. These results are in line with the analysis performed in the Copenhagen cohort showing that GOLD B patients had more severe dyspnea and significantly poorer survival than group C ones, in spite of a higher FEV1 level [5]. The same trend concerned the number of comorbidities, with a prevalence of comorbidities (more than two) in GOLD B and D patients. At an equal level of severity, management of COPD seems to be different in severe COPD patients with comorbidities, with more LABA and SABA in severe COPD, suggesting that comorbidities could increase respiratory symptoms. Moreover, prevention of exacerbations requires interventions beyond the lungs, including treatment of comorbidities such as gastro-esophageal reflux disease, reduction of cardiovascular risks, and management of dyspnea and anxiety [16].

LABA were prescribed the most in GOLD A patients, which is not in agreement with the guidelines GOLD 2011. This could be due to the high prevalence of symptoms like cough in this group, as symptoms included in the GOLD classification are based on dyspnea and exacerbations but not on cough. We cannot rule out that symptomatic GOLD A patients could represent a specific phenotype. Recently, Woodruff et col. described a subgroup of symptomatic patients with no criteria for COPD regarding lung function [17]. In addition, management of COPD differed according to the comorbidities that patients had, even if those with or without had the same level of severity. This was particularly the case for rehabilitation and vaccination which were more prescribed in symptomatic GOLD B and D patients who had comorbidities than in those without.

We expected to have a gradient in COPD severity, perhaps patients GOLD B should be called differently, as they seemed to be more severe than GOLD C.

The cluster analysis revealed five clusters: The cluster analysis showed five phenotypes of comorbidities: cluster 1 included cardiac profile; cluster 2 included less comorbidities; cluster 3 included metabolic syndrome, apnea and anxiety-depression; cluster 4 included undernutrition and osteoporosis and cluster 5 included bronchiectasis. Vanfleteren found 13 comorbidities in a sample of 213 COPD patients [10] with five comorbid phenotypes: less comorbidity, cardiovascular, cachectic, metabolic, and psychological. Four of our clusters are concordant, i.e. cardiovascular, cachectic, metabolic and less comorbidities. Nevertheless, all the clusters were more significantly associated with GOLD D and in less manner with GOLD B. This finding could explain the higher risk of mortality in GOLD B and D patients, as previously reported elsewhere [5]. In the same way, Divo et al. [9] also identified a number of modules in the comorbidity network, including a cardiovascular one, and a module characterized by mil-moderate airflow limitation and metabolic syndrome with high BMI, these two modules are concordant with our findings.

Our results show that while comorbidity in COPD is a complex issue, comorbidities contributed prominently to the clinical severity of our patients, and that management of their severe COPD differed according to whether they had comorbidities or not, at the same level of obstruction.

Our study has some limitations. First, comorbidities were recorded by pulmonologists; previous studies showed that comorbidities are underdiagnosed in real life. This could also be the case in our study for most comorbidity except for OSA, as OSA was diagnosed by a polysomnography performed by the same pulmonologist who diagnosed COPD. However, we think that this bias is limited in our study, as we found a significant correlation between the comorbidities declared and compliance with the treatment given for them (data not shown). Second, we cannot generalize these findings to patients with COPD in the general population, as our population was managed both by a general practitioner and a pulmonologist. Third, we performed a multivariable exploratory analysis in order to better describe the associations between the different comorbidities [18]. This type of analysis uses a statistical method that processes a large amount of information from heterogeneous variables in homogeneous groups. It is well known that various factors can influence the analysis and therefore the results: the choice of the methods, hierarchical or nonhierarchical, the determination of the number of clusters before the analysis, the choice of the variables included in the analysis, the correlation between the selected variables and the clinical judgment of the investigators. To limit the impact of the specific correlation between the variables, we first performed a principal component analysis. Then we used the scree plot of the eigenvalue, the Kaiser-Guttman criterion and the percentage of variance explained to determine the number of clusters.

Lastly, we cannot validate our clusters in terms of survival as the study was performed with inclusion criteria, or with systemic inflammation. Further analysis with survival data from these COPD patients would provide important information for validating these clusters of comorbidity.

## Conclusions

This study in a large clinic-based cohort shows that multimorbidity is common in patients with COPD, and that five comorbidity clusters can be identified. Patients GOLD B have more comorbidities than GOLD C.

The presence of comorbidities should therefore be included in any assessment of COPD severity. Further analysis is needed to validate these clusters in a future cohort.

## Additional file

Additional file 1: PALOMB English questionnaire (PDF 12 kb)

## Abbreviations

ATS/ERS: American Thoracic Society/European Respiratory Society
BODE: Bmi Obstruction Dyspnea Exercise
COPD: Chronic obstructive pulmonary disease
COTE index: Comorbidity index
FEV1: Forced expiratory Volume
GOLD: Global Obstructive Lung Disease Initiatives
ICS: Inhaled corticosteroids
LABA: Long acting bronchodilatator
LAMA: Long acting muscarinic antagonist
OSA: Obstructive Syndrome Apnea
SABA: Short acting bronchodilatator
