# Supplementary material for: Comorbidities and COPD severity in a clinic-based cohort
Source: BMC Pulm Med. 2018 Jul 16;18:117. doi: 10.1186/s12890-018-0684-7 (PMC6048834; doi:10.1186/s12890-018-0684-7)
Supplement: Supplementary file 1 — PALOMB English questionnaire (PDF 12 kb) [file 12890_2018_684_MOESM1_ESM.pdf]

Questions	Items
Date of visit	
ID number	
gender	
Date of birth	
Adress (postal code)	Outside France, Country
Occupation	1/Farmers 2/craftsmen<br>3/managers 4/intermediate occupation 5/employees<br>6/workers 7/retired 8/no professional activity
Habits	home / institution
Location of birth	City/department/country
Family status	couple/unmarried/divorced/ widow
Social Insurance	
Adressed by	
CLINIC	
Height/ Weight	(cm)/(Kg)
Blood pressure	(max/min mmHg)
Pulse	(nb pulsations/min)
Risk factor	
Smoking *Number pack years*	yes/no if yes, past smoker or active smoker
Minimal advice	Yes/no
e-cigarette	Yes/no
Environment	
Job exposure	
Genetic predisposition	alpha 1 antitrypsin deficiency
Clinical examination	
Purpose of the visit*	
Year of first symptoms	
Cough*	Yes/no
Phlegm*	Yes/no
Dyspnea/MRC	
Chest pain *	Yes/no
Exacerbations*	(nb/year)
Daily time of walking	(min)
Pulmonary examination	
Spirometry	
FEV1 (post bronchodilatator)*	(L)
LVC or FVC*	(L)
TPC	(% predicted)

**RV**	**(% predicted)**
**TLCO/VA**	**(% predicted)**
**IC**	**(% predicted)**
**Blood gases**	
**SaO2**	**(%)**
**PaO2**	**(mmHg)**
**PaCO2**	**(mmHg)**
Exercise	
**6 minutes walking test**	**(meters)**
**COMORBIDITiES**	
**OAS**	**Yes/no**
**Asthma**	**Yes/no**
**bronchiectasis**	**Yes/no**
**Cancer**	**Yes/no**
**Cardio-vascular**	
**Hypertension**	**Yes/no**
**Ischemic cardiopathy**	**Yes/no**
**Heart rhythm disorder**	**Yes/no**
**Left cardiac insufficiency**	**Yes/no**
**Atheroma**	**Yes/no**
**Vascular Stroke**	**Yes/no**
**Pulmonary hypertension**	**Yes/no**
**Arteriopathy**	**Yes/no**
**METABOLIC**	
**Diabetus**	**Yes/no**
**Dyslipidémia**	**Yes/no**
**Metabolic syndrome**	**Yes/no**
**Denutrition**	**Yes/no**
**Osteoporosis**	**Yes/no**
**Depression**	**Yes/no**
**Anxiety**	**Yes/no**
**Rhinitis/rhinosinusitis**	**Yes/no**
**Treatment**	
**COPD medication**	**Link with VIDAL database**
**Compliance**	**Good/moderate/low**
**Oxygen therapy**	**Long term /Nocturnal /during wlaking/short term**
**Non invasive ventilation**	**Yes/no**

**Continue Positive Pressure**	**Yes/no**
**Smoking cessation management**	**Yes/no**
**NRT use**	**Yes/no**
**Physiotherapy breathing**	**Yes/no**
**Therapeutic education**	**Yes/no**
**Respiratory rehabilitation**	**Yes/no**
**PREVENTION**	
**Influenza and pneumococcal Vaccinations**	**Yes/no**
**Psychological support**	**Yes/no**
**Exacerbations : action plan**	**Yes/no ; written/oral**
**Physical activity**	**Yes/no**

**Next visit**	**15 days/1month/3months/6months/1year**
